# Cost-Effectiveness of Fecal Microbiota Transplantation in the Treatment of Recurrent Clostridium Difficile Infection: A Literature Review

**DOI:** 10.7759/cureus.1599

**Published:** 2017-08-23

**Authors:** Leor T Arbel, Edmund Hsu, Keegan McNally

**Affiliations:** 1 University of Central Florida College of Medicine

**Keywords:** c. difficle, fecal microbiota transplantation, antibiotics, diarrhea, cost effectiveness

## Abstract

Clostridium difficile (*C. difficile*) is a common cause of antibiotic-­associated diarrhea (AAD), being responsible for 15­-25% of all AAD cases. The purpose of this literature review is to determine the cost-effectiveness of fecal microbiota transplantation (FMT) and how it compares in this regard to the standard treatments of choice for recurrent C. difficile infection (CDI). The review of the literature along with the evaluation of three comparative cost effective analyses yielded findings consistent with the view that FMT is the most cost-effective option in treating recurrent CDI. There are some (but considerably less) data indicating that FMT may be a cost effective strategy in treating initial CDI, as well. The superior cost-effectiveness of FMT as compared to the preferred standards of treatment for recurrent CDI suggest FMT use should become more integrated in routine clinical practice. Increased utilization of FMTs would allow for better control of this increasingly problematic disease as well as lower costs associated with its management.

## Introduction and background

Clostridium difficile [C. difficile] is a common cause of antibiotic-associated diarrhea [AAD], accounting for up to 25% of all AAD cases. This anaerobic Gram-positive bacillus forms exotoxins [toxin A and toxin B] and spores, which are found in the feces of infected patients [1]. Transmission of C. difficile occurs through contact with surfaces contaminated with feces. The patients at greatest risk for infection are the elderly [≥ 65 years old], especially those receiving care in hospitals or nursing homes [2]. The most significant risk factor, however, is a history of recent antibiotic exposure. The most frequently implicated antibiotics include clindamycin, fluoroquinolones, penicillins, and cephalosporins. The C. difficile infections [CDIs] arise most commonly in patients on prolonged antibiotic regimens as this destroys the normal flora of the gut [2-3]. The C. difficile then grows in place of the normal gut flora, producing the aforementioned toxins which damage the intestines and cause disease.

The disease caused by uncontrolled CDI ranges from mild diarrhea to pseudomembranous colitis with potentially fatal sequelae such as bowel perforation, toxic megacolon, and sepsis [1]. Furthermore, CDIs have been known to recur in 20-35% of patients treated for initial CDI and in up to 65% in patients treated for recurrent CDI [rCDI] using conventional treatment strategies [3-5].

In addition, due to factors such as the emergence of the hyper virulent BI/NAP1/270 strain and a growing population of susceptible older adults, the last decade has seen a dramatic upsurge in both the incidence and severity of CDI and rCDI [4]; this, in turn, has led to higher mortality, increased morbidity and longer hospital stays, ultimately culminating in a dramatic increase in the disease burden and healthcare costs associated with the condition [3]. Indeed, C. difficile is now recognized as the leading cause of nosocomial infection, and hospital costs associated with CDI in the United States are thought to exceed $3.2 billion per year [6]. The CDI recurrence is of particular concern as it accounts for a significant portion of this figure due to its association with longer hospital stays and more limited treatment options [6-7]. In light of these disquieting trends and the immense expenditures they portend, identification of the most cost-effective way to treat CDI and rCDI has become of paramount importance. Implementation of a more economical treatment strategy which delivers better health outcomes for affected patients would be optimal as it would simultaneously address the mounting burden of the disease and the staggering medical costs associated with CDIs.

Currently, there are a number of different treatment options available and their use varies by level of CDI severity [8]. Historically, patients presenting with mild symptoms often did not require treatment, as the normal gut flora could repopulate and successfully fight the infection on its own. Nowadays, however, most symptomatic C. difficile infections require treatment. The current standards for initial CDI proffer oral metronidazole for mild to moderate cases and oral Vancomycin in more severe cases [9]. When treating C. difficile infections with antibiotic regimens, it is important to take the antibiotics as prescribed in order to ensure adequate control of C. difficile populations. In patients who relapse and develop rCDI, a repeat course of the initial antibiotic therapy is recommended; an alternative option consistent with current guidelines is fidaxomicin, a macrolide antibiotic approved for use in CDI in 2011. For second relapses, tapering and pulsed oral Vancomycin is the treatment of choice, although fidaxomicin is again an acceptable alternative. For third and subsequent relapses, fidaxomicin is the standard medication, if it has not been used in the patient before [9]. Beyond that, guidelines on multiple recurrent CDI remain poorly defined. Fortunately, however, a number of promising investigational therapies for its treatment have emerged; these include rifaximin , probiotic therapy, monoclonal antibodies, tigecycline, nitazoxanide and fecal microbiota transplantation [FMT], the latter of which is sometimes referred to as “fecal bacteriotherapy” as it involves the transfer of feces from a healthy individual into the large intestine of a CDI affected patient in order to restore a normal gut microbiome [9-12]. 

Of these, FMT, in particular, has garnered considerable attention in recent years due to numerous accounts of its remarkable efficacy in treating rCDI and its relatively low-cost profile [13-15]. In fact, it has been hailed as the most cost effective treatment option, especially in the setting of rCDI [16-17]. Given these encouraging assertions and the growing need for more cost-effective solutions, an investigation of the extent to which economically sound cost-effectiveness analyses are made comparing FMT vs. standard treatments supporting. This notion is both timely and of practical importance to the future management of C. difficile associated disease.

Methods

We performed a literature search of comparative cost effectiveness analyses for fecal microbiota transplantations and current standards of treatment for rCDI using University of Central Florida OneSearch, which compiles results in order of relevance to search terms from numerous databases including Medical Literature Analysis and Retrieval System Online (MEDLINE), Cochrane Database of Systematic Reviews, Cochrane Central Register of Controlled Trials, CINAHL Plus, Health Technology Assessments and ScienceDirect. We applied the following criteria to reduce the level of results with limited applicability to evidence-based medicine. 

The following criteria were used to further narrow down papers published:

1) Published between January 2010 to January 2017

2) Studies with human participants/patients

3) Papers that assessed the cost effectiveness of FMT for CDI compared to preferred options for CDI (oral vancomycin, metronidazole, fidaxomicin)

4) Written in English

5) Search topic pertains to "cost effectiveness" and "fecal microbiota transplantation" or "fecal microbiota transplant" or "FMT" or "fecal transplant".

In order to properly assess the cost-effectiveness of the FMT treatment modality, the studies included in our review investigated both the benefits and the costs of FMT in the treatment of rCDI. Furthermore, to provide context for meaningful interpretation, incorporated analyses compared the same factors for FMT as for competing for treatment strategies, which consisted predominantly of regimens containing oral metronidazole and/or oral vancomycin that represent the current standards of treatment for rCDI. For additional context, a review of FMT efficacy [defined as resolution of disease] was examined in contrast to standard treatment efficacy per comparative randomized controlled trials performed.

Results

Resolution Of Disease With Fecal Microbiota Transplantation Compared To Standard Treatments

It is worth mentioning that randomized controlled trials comparing oral metronidazole to oral vancomycin reflect equal efficacy in treating patients with Clostridium difficile associated diarrhea and colitis [18]. For this reason, both are regarded as acceptable first line options for the initial treatment of CDI, although metronidazole is generally preferred for mild to moderate cases due to its lower cost and a lesser potential for inducing resistance while vancomycin is favored in cases of more severe illness [9, 11, 18-19]. If CDI relapses, the patient is typically treated with a repeated course of the initial antibiotic [9, 19]. If the patient relapses again, he/she may be given tapering and pulsed oral vancomycin with or without probiotics such as Saccharomyces boulardii. Only if the patient relapses a third time, an FMT will be offered to the patient per current guidelines [9, 20].

Interestingly, current reports suggest that standard antimicrobial treatment of C. difficile infection is associated with relatively poor cure rates. While historically, these antibiotics were associated with a nearly 90% cure rate, more recent publications suggest that up to 50% of the patients with CDI are either refractory to treatment or go on to develop recurrence of disease [21-22]. Indeed, recurrence of CDI has become an increasingly common and severe phenomenon following treatment with these agents [23]. It is perhaps unsurprising that these agents have also demonstrated underwhelming efficacy in resolving these relapses according to recent randomized controlled trial results [24-25]. Fecal microbiota transplantation [FMT], on the other hand, has shown excellent efficacy in treating rCDI, with reported disease resolution rates ranging from 81-94% [1, 3]. The mechanism by which FMTs are able to achieve this is believed to be through repopulation of Bacteroides and Firmicutes species, both of which are deficient among patients with rCDI. Ninty one results from randomized controlled trials comparing the resolution rates of FMT to conventional therapies in patients with rCDI are summarized in Tables 1-2.

**Table 1:** Fecal microbiota transplantation vs standard vancomycin regimen for the treatment of recurrent C. Difficile infection, 2014 randomized controlled clinical trial outcomes (Cammarota, et al.). A total of 39 patients enrolled in the trial between July 2013 and June 2014 (Female = 23, Male = 16, mean age 73 years). They were randomly assigned to one of the two treatment regimens: (1) Experimental, FMT via colonoscopy (following a brief regimen of oral Vancomycin) or (2) Active comparator, oral Vancomycin only (prolonged course). No patient refused the proposed treatment. The study was terminated at the one ­year interim analysis due to the overwhelming efficacy of the FMT regimen compared to oral Vancomycin; accordingly, additional patients were not recruited. Results at the one­year interim analysis showed significantly higher resolution rates of C. difficile infection following FMT than with Vancomycin treatment alone (90% vs 26%, P < 0.0001). Except for minor gastrointestinal (GI) complaints that resolved within the first 12 hours of donor feces infusion, no adverse events in the FMT arm of the study were noted. No adverse events specifically related to Vancomycin were reported in the oral Vancomycin harm either [25].

	*n*	F	M	Mean age	Number of patients cured	Percent of patients cured
FMT (by colonoscopy)	20	12	8	71	18	90%
Vancomycin (oral)	19	11	8	75	5	26%
Total	39	23	16	­	23	­

**Table 2:** Duodenal infusion of donor feces for recurrent clostridium difficile, 2013 randomized controlled clinical trial outcomes (Van Nood, et al.). A total of 43 patients were originally included in the study from January 2008 through April 2010. One patient was subsequently excluded upon further analysis, leaving 42 patients who was able to participate in the study protocol. Another patient in the Vancomycin only group opted to discontinue all medication and died 13 days after randomization without providing data on response. Thus, only 41 patients completed the study protocol. The patients were randomly assigned to receive nasoduodenal donor feces infusion, Vancomycin only, or bowel lavage + Vancomycin; the latter two groupings represent the comparators in this study. Analysis of the proportions of patients cured by the infusion of donor feces, standard vancomycin therapy, and standard vancomycin therapy plus bowel lavage revealed that donor­ feces infusion was statistically superior to either of the comparators (P < 0.001 for overall cure rates) [24].

	*n*	F	M	Mean age	Number of patients cured	Percent of patients cured
FMT (by nasogastric tube)	16	8	8	73	15	94%
Vancomycin (oral), 14 days	13	7	6	66	4	31%
Bowel lavage + Vancomycin (oral)	13	3	10	69	3	23%
Total	42	18	24	­	23	­

Cost Effectiveness Of Fecal Microbiota Transplantation Compared To Standard Treatments

As said, disease resolution rates represent only one way by which one may gauge the effectiveness of an intervention. Furthermore, for reasons beyond the scope of this analysis, it is not the preferred measurement of effectiveness in cost utility evaluations. Rather, it is the quality adjusted life years (QALY), a single figure that accounts for both the length and the quality of life gained with a particular intervention, which is favored for conveying treatment effectiveness in analyses of this variation [26]. The QALY is then interpreted in the context of the costs associated with delivering the treatment in order to arrive at a common denominator of cost/QALY. Studies comparing different treatment options which are not necessarily mutually exclusive often express results in cost/QALY [27-29].

In accordance with this convention, a comparison of the costs, QALY, and cost/QALY ratios for FMTs vs currently preferred treatment strategies was performed using the results from each of the studies incorporated in our review. Our key findings are summarized in Tables 3-4. The QALY figures reported were obtained directly from the studies and therefore may reflect differing inputs and assumptions. For reference, interventions that cost less than $50,000 to $60,000 per QALY gained are generally deemed reasonably efficient (in the United States) [27, 30].

**Table 3:** Results from cost ­effectiveness analysis of fecal microbiota transplantation for recurrent clostridium difficile infection (Varier, et al.). Varier, et al. (2015) designed a decision ­analytic simulation model based on inputs from published literature to assess the cost effectiveness of FMT compared to tapered Vancomycin in treating recurrent CDI. Their base case analysis showed that FMT was both. The study concluded that FMT may be a cost­ saving intervention [31].

	Cost	QALY	Cost/QALY
Fecal Microbiota Transplantation (FMT)	$1,669	0.242	$6,896.69
Vancomycin (oral)	$3,788	0.235	$16,119.15

**Table 4:** Results from cost-­effectiveness of fecal microbiota transplant in treating clostridium difficile infection (Zowall, et al.), with supplementary conversion of CAD to USD using 2011 exchange rate. Zowall, et al. study (2011) constructed a decision analytic model to compare strategies for the management of CDI in terms of their cost-­effectiveness. The strategies for comparison in their study were FMT vs current practice (Metronidazole and Vancomycin). The model was informed using data from four Canadian provinces (Quebec, Ontario, British Columbia and Manitoba). The model used an estimated recurrence rate of 10.4% with FMT treatment. The recurrence rate for antibiotic treatment was estimated as 25.3% and 35.9% for first and second recurrences, respectively. The results of their analysis are summarized above. The researchers concluded that FMT is associated with lower costs and higher QALY (and thus lower cost/QALY) than conventional antibiotic treatments. Accordingly, their conclusions endorse FMT as the dominant treatment strategy for the treatment of CDI [32].

	Cost (millions)	QALY	Cost/QALY	Cost in USD (millions)	Cost in USD/QALY
FMT (age ≥ 18y)	$253	11,941	$21,187.51	$255.90	$21,430.40
Current practice (age ≥ 18y)	$309	11,410	$27,081.51	$312.54	$27,391.97
FMT (age 18­-59y)	$43	3,016	$14,257.29	$43.49	$14,420.74
Current practice (age 18­-5y)	$49	2,928	$16,734.97	$49.56	$16,926.82
FMT (age 60­-79y)	$95	4,695	$20,234.29	$96.09	$20,466.26
Current practice (age 60-­79y)	$118	4,480	$26,339.29	$119.35	$26,641.24
FMT (age ≥ 80y)	$114	4,230	$26,950.35	$115.31	$27,259.31
Current practice (age ≥ 80y)	$142	4,002	$35,482.26	$143.63	$35,889.03

Incremental cost effectiveness ratio (ICER) is another index of cost-effectiveness that is commonly calculated in economic evaluations of health care decisions. ICER is similar to the cost/QALY ratio described above, except that ICER represents the difference between the cost of two possible interventions divided by the difference in their effects [33]. Because ICER is incremental and comparative in nature, it is ideal for ranking multiple interventions in settings in which options are mutually exclusive [28, 30]. A study of the year 2014 included in our review compared metronidazole, vancomycin, fidaxomicin, and FMT in the treatment of initial CDI using a decision analytic model; the outcome of interest was ICER, cost, and QALY and that was reported as well [34]. The key findings from this study are summarized in Table 5.

**Table 5:** Base case & sensitivity analyses of competing strategies for the management of recurrent clostridium difficile infection (Konijeti, et al.). Summary of findings from Konijeti, et al. (2014) decision analytic model­based study comparing four treatment strategies for recurrent CDI: A strategy was considered dominated if the preceding non­dominated alternative was both more effective and less expensive. ICER was calculated for fidaxomicin relative to the next non­dominated strategy (vancomycin). These results indicate that FMT by colonoscopy was the most cost­effective strategy in the base case analysis (scenario A), with an ICER of $17,016 compared to Vancomycin. However, FMT delivered by duodenal infusion (scenario B) and enema (scenario C) were found to be less cost­effective; thus, in scenarios B and D, where FMT could not be delivered by colonoscopy (or at all), initial oral Vancomycin was the preferred approach [34].

Scenarios	Cost			QALY	ICER	
A. All 3 pharmacologic treatment arms, FMT via colonoscopy (base case)						
Vancomycin	$2,912			0.8580		
FMT colonoscopy	$3,149			0.8719	$17,016	
Metronidazole	$3,941			0.8292	(dominated)	
Fidaxomicin	$4,261			0.8653	(dominated)	
B. All 3 pharmacologic treatment arms, FMT via duodenal infusion						
Vancomycin	$3,531			0.8484		
Metronidazole	$3,941			0.8292	(dominated)	
FMT duodenal infusion	$4,208			0.8553	$97,352	
Fidaxomicin	$4,628			0.8596	$98,443	
C. All 3 pharmacologic treatment arms, FMT via enema						
Vancomycin		$3,488	0.8485			
Metronidazole		$3,941	0.8292			(dominated)
FMT enema		$4,090	0.8543			$105,003
Fidaxomicin		$406	0.8597			$99,862
D. Only the 3 pharmacologic treatment arms alone (ie FMT unavailable)						
Vancomycin		$2,912	0.8580			
Metronidazole		$3,941	0.8292			(dominated)
Fidaxomicin		$4,261	0.8653			$184,023

## Review

Healthcare costs in the United States have become one of the most pressing economic concerns of the 21st century. Currently, it is estimated that the United States spends about 18% of the GDP on healthcare, leading the world in healthcare spending [35]. However, even with this increase in spending, the United States lags behind in life expectancy at birth and life expectancy at 65- 79 years. The life expectancy at birth in the United States is the lowest out of the top 18 countries in terms of healthcare spending [35]. Better and more cost effective management is of paramount concern for the healthcare industry as a whole; in depth, studies of both outcomes and cost effectiveness of new treatment options for establishing diagnoses like the case of FMT for recurrent CDI must be performed in order to more effectively manage patients and reduce the healthcare burden.

In our analysis of different studies of the cost effectiveness of fecal microbiota transplantation for the treatment of recurrent CDI, FMT has been associated with both better outcomes and as a more cost effective treatment.

While examining outcomes for recurrent CDI treatment using vancomycin and FMT [24-25], two studies have shown that both FMT by colonoscopy and FMT by duodenal infusion have resulted in significantly higher cure rates for patients with recurrent infection. By colonoscopy, the cure rates of infection were 90% for FMT vs. 26% for vancomycin in a 39 patient sample from July 2013 to June 2014 [25]. In a different study of rCDI treatment using a duodenal infusion of FMT, the cure rates of infection were 94% for FMT, 31% for Vancomycin, and 23% for Vancomycin + bowel lavage in a study of 42 patients from April 2008 to January 2010 [24]. Both studies were statistically significant (P < .0001 and P < .001 respectively) and showed that FMT could be a viable alternative to vancomycin treatment.

However, even with the viability of FMT as a treatment for rCDI, its cost effectiveness is paramount to the discussion as rCDI is associated with long and costly hospital stays [3]. Three separate studies involving the cost effectiveness of FMT demonstrated its advantages over traditional vancomycin treatment [31-32, 34].

The first study (represented in Table 3) analyzed costs, quality adjusted life years (QALY) and cost/QALY, all of which favored FMT vs. traditional vancomycin treatment (cost/QALY: $6,896.69 vs. $16,119.15) [31]. This demonstrated that not only is the procedure generally considered cheaper, it is also associated with better outcomes, and when combining both factors, FMT demonstrated far superior cost effectiveness. This study did demonstrate some limitations, however, the author’s own words a “lack of existing studies examining FMT from which to gather inputs” shows that more studies must be conducted in order to gather more accurate data regarding the true cost benefit of FMT [31].

Another study (represented in Table 4) also analyzing costs, QALY and costs/QALY demonstrated a similar result, showing that FMT was more cost effective than the current practice for all age groups 18 years or older [32]. For all age groups, cost/QALY of FMT vs. current practice (metronidazole and Vancomycin) was $21,430.40 vs. $27,391.97. This study concluded that FMT is associated with lower costs and the authors endorsed FMT as the dominant treatment strategy for treating recurrent CDI.

Lastly, we examined a study that used an incremental cost effectiveness ratio (ICER) to evaluate the cost effectiveness of multiple different treatment modalities for rCDI. These included metronidazole, Vancomycin, fidaxomicin and FMT [34]. Although the results do show that FMT by colonoscopy is the most cost effective treatment when all four treatments are compared, the study also showed that vancomycin was more cost effective if FMT is performed by duodenal infusion, enema, or when FMT is unavailable. This leads to some limitations in treatment, as FMT colonoscopy may not always be a viable option for the treatment.

Some limitations and stipulations must be applied to both our study and the current state of research into FMT. Our study was limited by the current amount of data on FMT and focused on cost-benefit analyses, which have a number of significant drawbacks. In some cases, cost-benefit analyses may not take into account all relevant health outcomes and variations in treatment across a diverse population with geography and access to care. As this is the case of the studies currently available for FMT, these studies are models of health-outcomes and cost and are not randomized controlled trials. The authors of the studies in this research do believe that the benefits of their models outweigh the disadvantages of these studies with regards to their inherent limitations.

Furthermore, because FMT is a developing treatment procedure, new evidence could come to light that is more compelling than preliminary results discussed here. A new randomized controlled trial sponsored by the North Shore University HealthSystem analyzing outcomes for FMT versus antimicrobial treatment for rCDI was verified on March 2016 and is currently undergoing recruitment [36]. This study and other randomized controlled trials (RCTs) in the future could help reveal some of the details regarding specific outcomes for FMT, which could then be incorporated into older and newer studies examining the cost-benefit ratio of FMT vs. standard treatment. Overall, because of the current state of research, we feel as if our current analysis of FMT reveals new insight into the possibility for FMT to become the mainstay of treatment for rCDI, based on both outcomes and cost-benefit, but new research must be conducted in order to determine how substantial that benefit may be. 

Though more compelling evidence may arise, FMT has already demonstrated greater efficacy with comparable or even lower costs than the conventional agents which comprise our current standards of care for recurrent CDI. Furthermore, in contrast to the limitations on patient acceptance predicted by the researchers, recent findings indicate that the vast majority of patients would be willing to receive FMT despite its inherently unappealing nature, especially if it were endorsed by a physician [17, 36-37]. The barrier though maybe an endorsement from a physician. For instance, it has been postulated that physicians, curiously enough, have been less willing to accept FMT as a therapeutic modality than their patients [38]. Ongoing and future research can address the limitations in the previous studies, particularly those stemming from a lack of sufficient data. This underscores the need for more multisite, adequately powered, high-quality studies comparing the costs and benefits of viable treatment modalities for recurrent C. difficile [39].

## Conclusions

At present, FMT is not a first line option in the treatment of adults with CDI. In fact, it is generally not a second or third line option either. Thus, FMT use is essentially restricted to treatment of CDI in individuals who have relapsed despite the first, second and third line therapy. Moreover, many facilities do not offer FMT as an intervention altogether. With this review, given the apparent readiness of patients to accept this as a treatment, there is evidently favorable cost effectiveness profile against a backdrop of rising healthcare costs and the increasing incidence and morbidity associated with this disease, it would be rational to expect a more widespread adoption of FMT in the treatment of rCDI than that which is currently observed. It stands to reason, then, that barriers to adoption unrelated to cost, efficacy or patient perception exist. In order to better characterize and ultimately address these concerns, further exploration of the impediments to broader FMT utilization is warranted. Since negative attitudes towards FMT among physicians would certainly hinder FMT integration in routine clinical practice, it may be worthwhile to study this subject in greater detail. Alternatively, it is plausible that logistical constraints, lack of awareness, and/or uncertainty regarding the route of administration are to be blamed. Even more generally, however, the epidemiologic and financial data suggest there is a need for continued generation and judicious review of evidence-based research on both preventative and novel interventions, FMT and otherwise, that may enable us to contain and combat this growing epidemic more effectively and efficiently.
